# Potential contribution of cereal and milk based fermented foods to dietary nutrient intake of 1-5 years old children in Central province in Zambia

**DOI:** 10.1371/journal.pone.0232824

**Published:** 2020-05-08

**Authors:** Justin Chileshe, Elise F. Talsma, Sijmen E. Schoustra, Karin J. Borgonjen-van den Berg, Ray Handema, Bas J. Zwaan, Inge D. Brouwer

**Affiliations:** 1 Laboratory of Genetics, Plant Sciences Group, Wageningen University and Research, The Netherlands; 2 Division of Human Nutrition and Health, Wageningen University and Research, The Netherlands; 3 Tropical Diseases Research Centre, Ndola, Zambia; 4 Department of Food Science and Nutrition, School of Agricultural Sciences, University of Zambia, Zambia; Università degli Studi di Milano, ITALY

## Abstract

Zambia is still facing undernutrition and micronutrient deficiencies despite fortification and supplementation programmes stressing the need for additional solutions. Fermented foods have the potential to improve nutrient intake and, therefore, could have an important role in food based recommendations (FBRs) to ensure adequate intake of nutrients for optimal health of populations. Secondary dietary intake data was used in Optifood, a linear programming software to develop FBRs, for children aged 1–3 and 4–5 years in Mkushi district of Zambia. Three scenarios per age group were modeled to determine FBRs based on: (1) FBRs based on local available foods (2) FBR and *Mabisi*, a fermented milk beverage, and (3) FBR with *Munkoyo*, a cereal fermented beverage. The scenarios were compared to assess whether addition of *Mabisi* or *Munkoyo* achieved a better nutrient intake. FBRs based on only locally available non-fermented foods did not meet ≥70% of recommended nutrient intake (RNI) for calcium, fat, iron and zinc, so-called problem nutrients. The addition of *Munkoyo* to the FBRs did not reduce the number of problem nutrients, but after adding *Mabisi* to the FBR’s only iron (67% of RNI) in the 1–3 year age group and only zinc (67% of RNI) in the 4–5 year age group remained problem nutrients. *Mabisi*, a fermented milk product in combination with the local food pattern is a good additional source of nutrients for these age groups. However, additional nutrition sensitive and cost-effective measures would still be needed to improve nutrient intake, especially that of iron and zinc.

## Introduction

Undernutrition remains a severe public health problem in Zambia where 40% of the children under the age of five years are stunted, 15% have underweight and 6% are wasted [1]. Undernutrition in children leads to reduced growth, cognitive development impairment, greater susceptibility to infections and higher risk of mortality [2, 3]. Micronutrient deficiencies are also high in Zambia with 60% of the children between 6–59 months old suffering from anaemia which may result from iron deficiency among others and 54% of them affected by vitamin A deficiency [4, 5]. Different strategies and interventions exist to prevent or reduce undernutrition and micronutrient deficiencies. These include food-based strategies such as dietary diversification and food fortification, as well as nutrition education, public health and food safety measures, and supplementation [6]. Fortification and supplementation of micronutrients are seen as short and medium term solutions but are more expensive, and adherence might be low if intensive stimulation programs are not put in place [7]. Despite fortification of sugar with vitamin A and salt with iodine and supplementation implemented to reduce micronutrient deficiencies for more than two decades, Zambia is still facing undernutrition and micronutrient deficiencies stressing the need for additional solutions [8].

In Zambia there are a number of nutritionally dense foods locally available that can potentially be utilised to alleviate these nutrition problems [9]. The formulation, testing and implementation of food based recommendations (FBRs) can contribute to improve the nutrient intake, especially when these local nutrient dense foods are incorporated [10]. Such FBRs facilitate the formulation of Infant and Young Child Feeding (IYCF) practices being identified as one of the most effective public health interventions to improve young child survival in developing countries [11]. Especially in young children, the composition of diets and quality of foods consumed by populations have a direct impact on their health and wellbeing and thus FBRs are important in providing population-level and context specific guidance on consumption of appropriate foods to meet their nutritional needs [11].

Optifood is a linear programming software that allows formulation of FBRs based on the best combinations of local foods to optimize nutrient intake and model the extent to which these can supply nutritionally adequate diets [12–16]. In previous studies conducted with intake data from children of 6 months to 23 months old, gaps in intake of specific nutrients have been found especially that of calcium, iron and zinc [14–17]. Adding recommendations on increase of intake of animal source foods can potentially improve intake of calcium, iron and zinc but consumption of such foods in Zambia is low where diets are predominantly plant-based. In plant-based diets, preparation methods such as fermentation can be a method to make nutrients more bioavailable [18]. Phytates are present as storage compounds of phosphorous in cereals as complexes with metal cations such as iron, zinc and calcium. Phytates can be degraded by fermentation, in which microbial activity lowers the pH providing an optimum environment for enzymatic degradation by phytase leading to an increase in the cations making them bioavailable [19]. Fermentation is also beneficial in dairy products, as this process helps to convert lactose into more digestible components hence making milk more tolerable [20]. More generally, fermentation also makes micronutrients more bioavailable especially in plant based foods, gives food longer shelf life and contributes to a healthy ecology of intestinal bacteria which promotes general health, through fermenting bacteria (usually lactic acid bacteria) that serve as probiotics [18, 21–24]. Also fermentation of dairy products increases the amounts of micronutrients such as folate [25], vitamin B2 [26] and vitamin B12 [27] among others and are made more bioavailable.

Fermented foods traditionally are an important part of the diet in many countries and are now being advocated for inclusion in food based recommendations for regular consumption in some countries [28]. Zambia has a wide range of local fermented foods similar to western yoghurt, wine, and beer [29]. A number of traditional non-alcoholic fermented beverages are available such as *Mabisi* and *Munkoyo* which are consumed by all age groups [30]. *Munkoyo* is a fermented beverage made from maize porridge with Rhynchosia venulosa *(Munkoyo)* roots added whereas *Mabisi* is a fermented milk product made by allowing raw milk to ferment at ambient temperature in containers such as buckets [30, 31].

The prevailing levels of undernutrition among children in Zambia are severe especially stunting which is an indication of long term deficits of the quantity and quality of food. To combat undernutrition, there is urgent need to find possible solutions of improving nutrient intake. This study used linear programming to explore secondary dietary intake data of children, 1–5 years of age, in Mkushi, Zambia to assess the potential effect on nutrient adequacy of adding traditional fermented foods (*Mabisi* and *Munkoyo*) into FBRs. This is with a view to identify nutrient gaps and suggest food combinations the local diets can come as close to filling as possible with addition of fermented foods. This secondary dietary intake data was initially collected to determine maize intakes and vitamin A intakes with a view to introducing bio-fortified orange maize.

## Methods

### Study design

This study was based on cross-sectional dietary intake data collected previously with children in a rural, maize consuming population in Mkushi (Central Province, Zambia) [32]. Data was collected during harvest/early post-harvest season (May–June 2009) using the 24-hour recall method and was used to model weekly food based recommendations for children aged 1–5 years, with and without inclusion of *Mabisi* or *Munkoyo*. The harvest/early post-harvest season was chosen because the initial study was designed to capture data for the period of plenty and going into the lean period to determine the maize intakes and vitamin A intakes with a view to introducing bio-fortified orange maize.

The initial study was approved by the Tropical Diseases Research Centre (TDRC) Ethics review committee (Ndola, Zambia) reference number TDRC/ERC/0705/0409 and the International Food Policy Research Institute (Washington, DC, USA) Institutional review board [32]. All the data were fully anonymized before accessing them and the ethics committee waived the requirement for informed consent for the present study.

### Subjects

The original study assessed dietary intake of 320 children in Mkushi [32] and included children aged 6–59 months, for whom parents gave written informed consent and were residing within the project catchment area were included into the study. Children were excluded based on the following criteria: being outside the age group 6–59 months; if parents did not give consent; children not residing in the catchment area as defined by the project; severely malnourished children (WAZ or WHZ <-3 SD, based on anthropometric measurements and WHO growth reference data); and children with severe anemia (Hb <7.0 g/dl).

From this data set we selected two groups of non-breastfed children aged 1–3 years (n = 156) and 4–5 years (n = 65) for Optifood programming, based on the different recommended nutrient intakes of these two age groups. Breastfed children were excluded because of a too small sample size (n = 15).

### Dietary intake assessment

The dietary intake data was collected using the multi pass 24 hour recall as described elsewhere [33]. To collect the dietary intake data [32], the mothers of the children were asked to recall all foods and beverages consumed by their children, including amounts, during 24-hours of the previous day. Food portion sizes were estimated using examples of real foods, scaled photographs, or in volumes using standardized measuring spoons and cylinders carried by interviewers, and calibrated modelling clay. Information on feeding habits including plate sharing during meal times by children or family members, and on foods and quantities consumed outside of the home was collected. To convert portion sizes recorded in volumes to gram weight equivalents, a local conversion table was developed. Grams of ingredients consumed from the composite dishes was derived from the recipe data collected during the recall study or from standard recipes compiled prior to the initial study. Nutrient intake calculations were based on the food composition tables (FCT) developed by the initial study[32] [33].

### Data preparation

Data from the 24 hour recalls was used to generate model parameters for Optifood using Excel and Access 2010 (Microsoft Corporation) [34]. Model parameters per age group were defined as follows: (I) A list of non-condiment foods and drinks consumed by ≥5% of the children during the recall period to ensure the commonly consumed foods are included; (II) The serving size of each food was defined as the medium serving size in grams per day for all children who consumed that particular food; (III) The minimum and maximum number of servings per week for each food group and food subgroup defined as the 10^th^ and 90^th^ percentiles of the serving counts respectively. The maximum and minimum number of servings per food within a food subgroup was estimated based on percentage of children who consumed the food [15, 16, 35, 36]. In the initial study only 2 children in the 4–5 age group were reported to have consumed *Mabisi* with the volume similar to that of tea consumed and thus the serving size/day for *Mabisi* in the two age groups were estimated based on the tea serving volume/day; (IV) Staple foods were identified as foods belonging to the food groups’ grain and grain products or starchy roots. Snacks were defined as foods consumed only in between meals. The type of meal (snack or staple) was determined based on the nature of the food and time of the food consumption.

Thirteen nutrients were selected for analysis of nutrient adequacy including total fat, total protein, calcium, vitamin C, thiamin, riboflavin, niacin, vitamin B6, folate, vitamin B12, vitamin A as RAE, iron and zinc using the FAO/WHO RNIs [37].

Energy constraints were used to model FBR’s that ensured average energy requirement for the two age groups, by using mean body weight obtained from the initial study for each age group [38]. Dietary patterns in Zambia like in other developing countries are often rich in plant based foods and are high in Phytates, thus low bioavailability of iron (RNI: 11.6 mg/day assuming 5% bioavailability) and zinc (RNI: 8.3 mg/day assuming 15% bioavailability) were taken into account [39, 40].

The FCT used for this study included nutrient values for *Mabisi* (FCT as Sour milk). Nutrient values for *Munkoyo* were based on a composited recipe consisting of maize meal and water. The World Health Organization (WHO) estimated that the maximum iron and zinc bioavailability in maize based diets ranges between 10 and 15% [41]. Fermentation of maize based foods or beverages improves bioavailability of iron and zinc mainly by the breakdown of Phytates and is estimated to result in a 5% increase in bioavailability for iron, but not clear for zinc [42]. The values of iron and zinc for *Munkoyo* in the FCT were therefore increased by 5% to 10% and 20% respectively to take into account the increase in bioavailability through fermentation.

### Analysis in Optifood

All analyses were carried out with Optifood program version 4.0.9.0 using a three module approach, based on linear programming to design population specific weekly FBRs. Per target group, three scenarios were modelled to develop FBR, namely: a) local foods without *Munkoyo* or *Mabisi*, b) local foods with *Munkoyo* included and c) local foods with *Mabisi* included. The data analysis was done by the lead author and checked by other authors as quality control at every stage from data preparation in Microsoft excel and in Microsoft access to importing the data into Optifood. Independent analysis was performed by a second person and the results were compared. The linear programming analyses used in this study have been described in detail by others [10, 15, 35, 36]. In summary, for each scenario in each target group data was checked by running module 1 (to set up model parameters) to ensure that model parameters were producing realistic diets with energy contents within a sufficient range to allow for modelling. An expert who was familiar with local dietary patterns then examined the foods selected in these 16 optimized 7-day diets to decide whether at least some individuals from the population could consume them.

Module 2 (to identify food based recommendations) was run for each of the three scenarios and for each age group to develop two optimized diets called the food pattern diet and no-food pattern diet. The food pattern diet is the best diet with minimized deviations from RNIs within the target population’s average food pattern. The no-food pattern diet is the best diet with minimized deviations from RNIs deviating from the average food pattern while remaining within lower and upper food group constraints. Module 2 was used to identify the nutrient dense food (sub) groups that were likely to improve nutrient adequacy and needed to be tested in module 3. Foods that contributed at least 5% to any of the nutrients were identified as nutrient dense foods. [16, 35, 36].

In Module 3 (to test alternative sets of FBRs to select the best dietary recommendations for the target population) diets were modelled for all three scenarios in both target groups, two 7-day diets per nutrient (i.e. in total 26 diets) were modelled of which 13 maximised (best-case scenario) and 13 minimized the nutrient content of the diet, for one nutrient (worst-case scenario), by preferentially selecting respectively the lowest and highest nutrient dense foods for that specific nutrient.

In step (i) of module 3, a no recommendation diet was run for each scenario to identify problem nutrients. Problem nutrients were defined as nutrients that were less than 100% of RNI in the best-case scenario (maximized diet) of module 3 diet modelled without FBR constraints [16, 35, 36].

**Fig 1:**
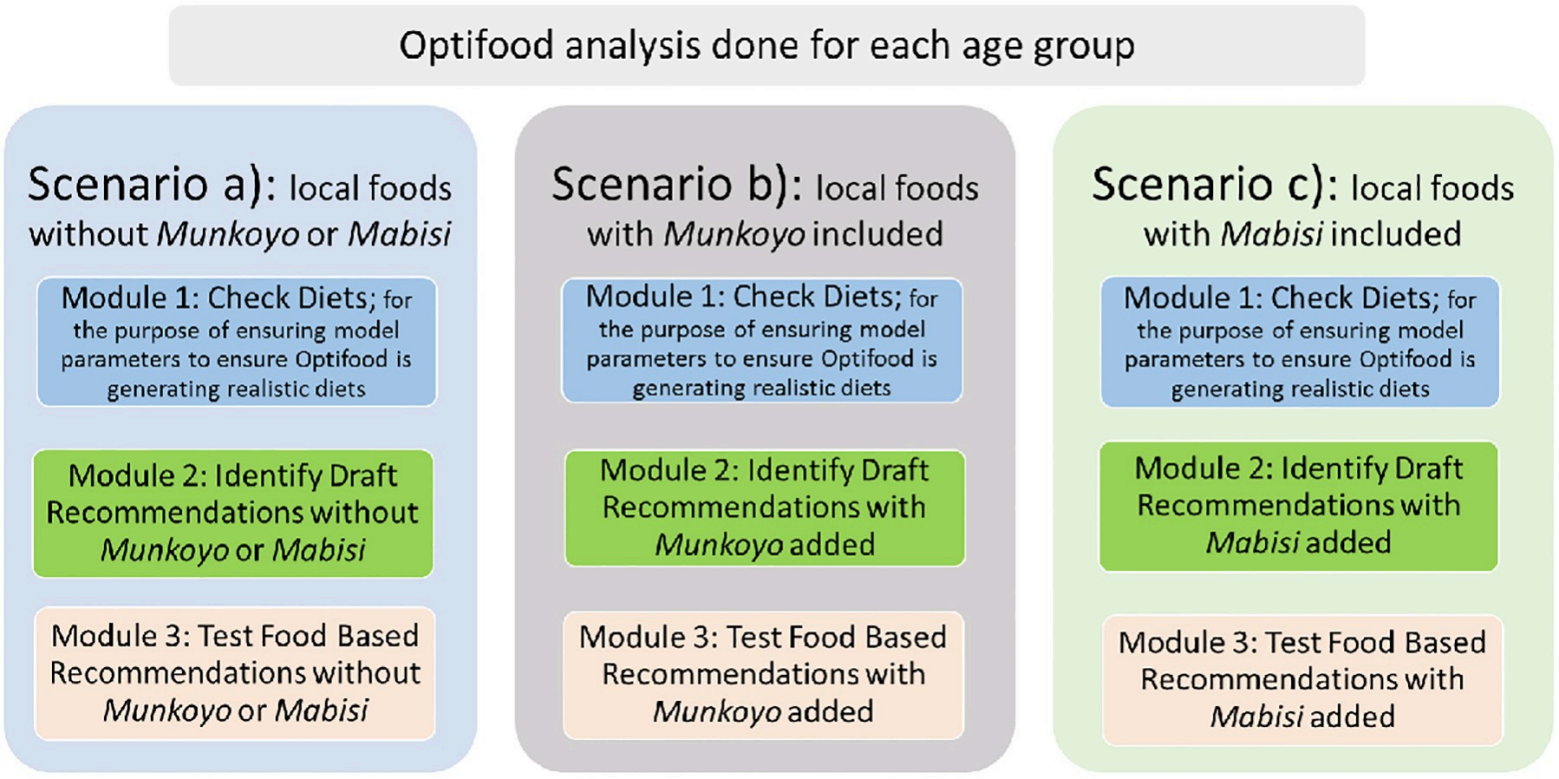
Graphical representation of Optifood analysis. Each age group was done separately and for each age group three scenarios were done. For each scenario, the three modules were applied to determine the FBRs.

In step (ii) of module 3, food groups with weekly servings above zero obtained in module 2 best food pattern diet, and nutrient dense foods and their accompanying food (sub) groups identified in module 2 were tested individually and were combined in step (iii) of module 3, the final FBRs per scenario in each target group was selected based on the combination of foods and food (sub) groups that covered 70% of the RNI in the worst case scenario for most nutrients, minimizing the deviation from the local food pattern as much as possible. The final FBRs were compared between the three scenarios. The outline of our approach of the modules for each scenario are presented in Fig 1.

## Results

Data of 156 children in the 1–3 year age group with an average age of 2.8 years (SD = 8.1) and 65 children in the 4–5 year age group with an average age of 4.3 years (SD = 3.7) were used in this study. Girls represented 47% (n = 74) in 1–3 age group and 61% (n = 45) in 4–5 age group.

A total of 164 foods (with 28 foods consumed by ≥5% of children) were consumed by children in 1–3 year group and 115 foods (with 31 foods consumed by ≥5% of children) were consumed by children in the 4–5 year group. The most commonly consumed foods in both age groups included vegetable oil, onions, tomatoes, maize flour, and rape leaves as a pro-vitamin A rich source green leafy vegetable. Maize meal (medium serving per day was 242g for 1–3 year olds and 255g for 4–5 year olds) was consumed in high amounts compared to other foods. The smallest serving sizes were for white sugar (14g/day) for the 1–3 year olds and for brown sugar (20g/day) for the 4–5 year olds. The consumption frequencies of the foods varied from 0 to 14 times per week. An overview of all the foods, their corresponding serving sizes and consumption frequencies used for analysis is shown in Table 1 for each of the two age groups.

**Table 1:** Foods and food (sub) groups consumed by children in the two age groups (1–3 years old and 4–5 years old) also describing the percentage of children who consumed a particular food, the serving size with the minimum and maximum number of servings per week.

Food group and food sub group^1^	Food	1–3 year olds				4–5 year olds			
		% children consuming	Serving Size (g)^2^	Min # serves/week^3^	Max # serves/week^4^	% children consuming	Serving Size (g)^2^	Min # serves/week^3^	Max # serves/week^4^
**Added fats**	Vegetable oil	80	24	0	7	81	26	0	7
**Added sugars**	Sugar, all grades, raw	10	22	0	7				
	Sugar, brown, raw	19	19	0	7	17	20	0	7
	Sugar, white, raw	13	14	0	2	13	41	0	2
	Sugarcane, raw					5	46	0	2
**Bakery & breakfast cereals**	Bun, bread, plain	5	98	0	3				
**Bread, wheat, refined flour, baked**	Fritters	7	87	0	7	7	78	0	7
	Bread, wheat, refined flour, baked	6	34	0	4				
**Beverages (non-dairy or blended dairy)**	Carbonated soft drink (soda)	7	208	0	7	6	208	0	7
	Fruit flavour drink,	6	242	0	7				
	Tea, brewed					11	193	0	7
**Composites (mixed food groups)**	Soup, Beef,					6	34	0	7
	Soup, Fish	8	24	0	7	6	34	0	7
**Fruits**	Banana, All Types	15	69	0	7	16	77	0	7
**Grains & grain products**	Maize Flour, white variety, breakfast, boiled	17	144	0	7	13	255	0	7
	Maize Flour, White Variety, Roller, boiled	88	166	7	7	87	214	0	14
**Legumes, nuts & seeds**	Beans, Lusaka, boiled	8	28	0	7	6	61	0	7
	Beans, navy, boiled	27	56	0	7	22	46	0	7
	Beans, pinto, boiled	11	75	0	7	12	60	0	7
	Groundnuts, Powder, boiled	39	42	0	7	24	21	0	7
	Groundnuts, Dried, raw	6	25	0	7	6	51	0	7
	Groundnuts, Dried, roasted	20	54	0	7	14	54	0	7
	Groundnuts, in-shell, fresh boiled					6	115	0	4
	Cowpeas, Dried, raw, boiled					7	74	0	7
**Meat, fish & eggs**	Egg, Chicken, Whole, boiled					6	33	0	7
	Fish, Kasepa, boiled	17	42	0	7	29	45	0	7
	Fish, Tilapia, boiled	6	23	0	7				
	Fish, Kapenta, boiled	10	64	0	7	7	55	0	4
**Starchy roots & other starchy plant foods**	Sweet potato, Yellow, boiled	59	231	0	7	52	257	0	7
**Vegetables**	Onion, boiled	98	26	7	7	98	26	2	7
	Tomato, boiled	98	92	7	7	98	101	8	8
	Rape Leaves (kale), boiled	52	56	0	7	51	85	0	7
	Pumpkin Leaves, boiled	20	54	0	7	14	80	0	7
	Cabbage, Green, boiled	26	46	0	7	27	53	0	7
	Sweet potato Leaves, boiled					9	41	0	7
	Pumpkin, Fresh, Boiled					7	163	0	7
**Fermented beverages**	*Munkoyo*		183	0	7		193	0	7
	*Mabisi*		183	0	7		193	0	7

^1^Food groups and food subgroups as defined by Optifood programme.

^2^Values are median serving sizes of raw edible portions when consumed on the basis of 24-hour recalls.

^3^Minimum frequencies were calculated on the basis of the 10^th^ percentile of distribution of the serving counts with consideration of proportion consuming each food within each food group.

^4^Maximum frequencies were calculated on the basis of 90^th^ percentile distribution of the serving counts with consideration of proportion consuming each food within each food group.

### Linear programming

Diets produced in Module 1 (to set up model parameters) were feasible and no changes in model parameters were needed. The number of nutrients reaching ≥100% of RNI in the two best diets of the three scenarios for each age group as analysed in module 2 (to identify food based recommendations), are shown in Table 2. For 1–3 years old children, 8 nutrients for the average food pattern and 10 nutrients for best food pattern reached ≥100%, with no differences whether in- or excluding *Mabisi* or *Munkoyo*. For 4–5 years old children, the average food pattern diet covered >100% RNI for 5 nutrients whether or not including *Mabisi*, however, including *Munkoyo* increased number of nutrients covering ≥100% RNI to 6. For this age group, 11 nutrients reached ≥100% RNI for the best food pattern without differences with or without *Mabisi* or *Munkoyo*. Fat, calcium and zinc were identified as problem nutrients for this age group. In module 2, food groups that included grains & grain products and vegetables; food sub groups that included nuts/seeds & unsweetened products, cooked beans/lentils/peas and small whole fish with bones; and rape leaves as a single food were identified as nutrient-dense foods to be included to develop food based recommendations in module 3 (to test alternative sets of FBRs to select the best dietary recommendations for the target population).

**Table 2:** Nutrient composition of the two optimized diets (Average food pattern and the best food pattern) for each of the three scenarios for the two age categories in module 2.

Nutrient	% of RNI^¥^ Covered for the 1–3 year olds						% of RNI^¥^ Covered for the 4–5 year olds					
	Diet without *Munkoyo* and *Mabisi*		Diet with *Munkoyo*		Diet with *Mabisi*		Diet without *Munkoyo* and *Mabisi*		Diet with *Munkoyo*		Diet with *Mabisi*	
	Average Food Pattern^#^	Best Food Pattern*	Average Food Pattern^#^	Best Food Pattern*	Average Food Pattern^#^	Best Food Pattern*	Average Food Pattern^#^	Best Food Pattern*	Average Food Pattern^#^	Best Food Pattern*	Average Food Pattern^#^	Best Food Pattern*
Protein	256	339	256	339	255	268.3	166	292	166	292	166.	296
Fat	75	67	75	67	75	69	88	73	88	73	88	56
Calcium	35	48	35	48	35	98	27	48	27	48	27	98
Vitamin C	316	312	316	312	316	310	399	438	399	438	399	438
Thiamin	116	112	116	112	115	108	82	125	82	125	82	108
Riboflavin	71	100	71	100	71	115	76	100	76	100	76	132
Niacin	193	515	193	515	187	100	63	141	63	141	63	100
Vitamin B-6	154	183	154	183	154	151	133	189	133	189	133	186
Folate	153	130	153	130	153	136	55	115	54.9	115	55	101
Vitamin B-12	183	1170	183	1170	169	100	23	100	23	100	24	133
Vitamin A RAE	97	101	97	101	97	113	133	163	133	163	133	174
Iron	68	88.4	68	88	67	83	58	100	58	100	58	100
Zinc	64	71	64	71	63	75	46	66	47	66	47	72
Nutrients ≥100% RNI	8	10	8	10	8	10	5	11	6	11	5	11

^#^Average food pattern was defined as best diet within average food pattern closest to the median food pattern of the population.

*Best food pattern was defined as the best diet deviating from average food pattern but constrained by the minimum and maximum serving per week.

^**¥**^RNI is the Recommended Nutrient Intake

In module 3 (i), fat, calcium, iron and zinc were identified as problem nutrients (reaching <100% RNI) in the best case scenario diet for the 1–3 year old group and fat, calcium and zinc for the 4–5 year old group. Testing food (sub) groups identified as nutrient dense in module 2, individually and combined (in module 3ii and iii), resulted in an increased coverage of the problem nutrients but none reached ≥70% RNI, and, hence, remained as problem nutrients (Table 3). Testing with inclusion of *Munkoyo* did not change these results (Table 4), however, inclusion of *Mabisi* resulted in fat, calcium and zinc disappearing as problem nutrient as their intake reached ≥70% RNI. Only iron remained as problem nutrient (Table 5).

**Table 3:** Shows Food based recommendations for scenario 1 with only local foods for the 1–3 year old group.

	Analysis	Nutrients as % of RNI^a^												
		Protein %	Fat %	Calcium %	Vitamin C %	Thiamin %	Riboflavin %	Niacin %	Vitamin B-6%	Folate %	Vitamin B-12%	Vitamin A RAE %	Iron %	Zinc %
	**module 2 optimized food patterns for the local**													
	Optimised Diets—Worst-case-scenario	255.8	74.7	34.6	315.9	115.6	71.1	192.8	154.5	153.3	182.8	97.2	67.6	63.5
	Optimised Diets—Best-case-scenario	338.6	67.2	48.4	312	112.4	100	514.7	183	129.8	1170.4	101.3	88.4	70.9
	**Module 3 Phase i (Without Recommendations)**													
	No Recommendation Diet—worst case scenario	106.7	24.4	6.1	38.7	55.9	33.3	41.8	77	27.5	3.7	0	37.2	37.7
	**Module 3 ii worst case scenario nutrient level for 6 single alternative sets of recommendations**													
1	Grains & grain products 7 servings	106.7	24.4	6.1	38.7	55.9	33.3	41.8	77	27.5	3.7	0	37.2	37.7
2	Small whole fish without bones 3 servings	194.5	39.1	12.3	39.8	61.6	59.2	365.1	110.4	29.4	873.9	4.1	41.9	44.5
3	Rape leaves (kale) 7 servings	118.8	24.4	20.7	262.7	66.3	46.9	50.6	107	37.3	4.3	95.3	44.8	40
4	Vegetables 21 servings	110.4	24.4	9.5	52.5	59.7	36.6	43.2	87	28.1	4.2	0.5	38.9	38.6
5	Nuts, seeds 3 servings	124.1	38.2	7.2	38.7	65.7	34.3	62.3	83.6	42.7	3.7	0	40.1	41.3
6	Cooked beans, lentils, peas 3 servings	125.4	24.4	10.1	38.7	57.3	34.6	41.8	80.3	46.1	3.8	0	47	42.6
	**Module 3 iii worst case scenario nutrient level for the combined single alternative sets to form FBRs**													
	1+2+3+4+5+6^b^	254.5	55.4^c^	34^c^	263.8	91.5	78.6	396.7	151.6	77.6	874.6	99.4	65.1^c^	56.8^c^

RNI–

^a^Recommended Nutrient Intake;

^b^Final FBRs—weekly diets modelled by combining the single food (sub) groups and single foods from module 3 b;

^c^Problem nutrients—nutrients that could not reach 70% of RNI

**Table 4:** Shows food based recommendations for scenario 2 based on local foods with *Munkoyo* added for the 1–3 year old group.

	Analysis	Nutrients as % of RNI^a^												
		Protein %	Fat %	Calcium %	Vitamin C %	Thiamin %	Riboflavin %	Niacin %	Vitamin B-6%	Folate %	Vitamin B-12%	Vitamin A RAE %	Iron %	Zinc %
	**module 2 optimized food patterns for the local**													
	Optimised Diets—Worst-case-scenario	255.8	74.7	34.6	315.9	115.6	71.1	192.8	154.5	153.3	182.8	97.2	67.6	63.5
	Optimised Diets—Best-case-scenario	338.6	67.2	48.4	312	112.4	100	514.7	183	129.8	1170.4	101.3	88.4	70.9
	**Module 3 Phase i (Without Recommendations)**													
	No Recommendation Diet—Worst case scenario	106.7	24.4	6.1	38.7	55.9	33.3	41.8	77	27.5	3.7	0	37	37.7
	**Module 3 ii worst case scenario nutrient level for 7 single alternative sets of recommendations**													
1	Grain & grain products 7 servings	106.7	24.4	6.1	38.7	55.9	33.3	41.8	77	27.5	3.7	0	37	37.7
2	*Munkoyo* 5 servings	107.1	26.1	6.5	38.7	64	42.6	46.1	81.8	31.4	5.1	0	37	41.5
3	Vegetables 21 servings	110.4	24.4	9.5	52.5	59.7	36.6	43.2	87	28.1	4.2	0.5	38.7	38.6
4	Rape leaves (kale) 7 servings	118.8	24.4	20.7	262.7	66.3	46.9	50.6	107	37.3	4.3	95.3	44.6	40
5	Small whole fish without bones 3 servings	194.5	39.1	12.3	39.8	61.6	59.2	365.1	110.4	29.4	873.9	4.1	41.9	44.5
6	Nuts, seeds 4 servings	131.5	43.5	7.7	38.7	69.5	35.1	69.5	85.8	48.5	3.7	0	41.4	42.6
7	Cooked beans lentils peas 3 servings	125.4	24.4	10.1	38.7	57.3	34.6	41.8	80.3	46.1	3.8	0	47	42.6
	**Module 3 iii worst case scenario nutrient level for the combined single alternative sets to form FBRs**													
	1+2+3+4+5+6+7^b^	265.7	63.7^c^	35.6^c^	263.9	106.4	89.9	408.9	159.2	88.5	876	99.4	67.1^c^	62.4^c^

RNI–

^a^Recommended Nutrient Intake;

^b^Final FBRs—weekly diets modelled by combining the single food (sub) groups and single foods from module 3 b;

^c^Problem nutrients—nutrients that could not reach 70% of RNI

**Table 5:** Shows Food based recommendations for scenario 3 based on local foods with *Mabisi* added for the 1–3 year old group.

	Analysis	Nutrients as % of RNI^a^												
		Protein %	Fat %	Calcium %	Vitamin C %	Thiamin %	Riboflavin %	Niacin %	Vitamin B-6%	Folate %	Vitamin B-12%	Vitamin A RAE %	Iron %	Zinc %
	**module 2 optimized food patterns for the local**													
	Optimised Diets—Worst-case-scenario	255	74.7	35.3	315.9	115.5	71.4	187.5	154.1	153.3	169.6	97.4	67.5	63.6
	Optimised Diets—Best-case-scenario	268.3	69.4	98.7	310.4	108.5	115.2	100	151.4	135.9	100	112.8	83.2	75.2
	**Module 3 Phase i (Without Recommendations)**													
	No Recommendation Diet—worst case scenario	106.7	24.4	6.1	38.7	55.6	33.3	41.8	77	27.5	3.7	0	36.7	37.7
	**Module 3 ii worst case scenario nutrient level for 7 single alternative sets of recommendations**													
1	Grains & grain products 7 servings	106.7	24.4	6.1	38.7	55.6	33.3	41.8	77	27.5	3.7	0	36.7	37.7
2	Rape leaves (kale) 7 servings	118.8	24.4	20.7	262.7	66.1	46.9	50.6	107	37.3	4.3	95.3	44.6	40
3	*Mabisi* 7 servings	150.6	42.1	64.3	38.7	58.3	85.7	43.8	88.9	31.7	93	16.9	37.3	50.1
4	Nuts, seeds 4 servings	131.5	43.5	7.7	38.7	69.5	35.1	69.5	85.8	48.5	3.7	0	41.4	42.6
5	Cooked beans lentils peas 3 servings	125.4	24.4	10.1	38.7	57.3	34.6	41.8	80.3	46.1	3.8	0	47	42.6
6	Vegetables 21 servings	110.4	24.4	9.5	52.5	59.5	36.6	43.2	87	28.1	4.2	0.5	38.6	38.6
7	Small whole fish without bones 2 servings	179	36.4	11.1	39.6	60	54.7	311	104.7	28.8	728.9	3.4	40.9	43.2
	**Module 3 iii worst case scenario nutrient level for the combined single alternative sets to form FBRs**													
	1+2+3+4+5+6+7^b^	295.3	78.1	92.7	263.7	101.4	129.5	352.8	161.2	88.7	818.9	115.7	67.1^c^	70.1

RNI–

^a^Recommended Nutrient Intake;

^b^Final FBRs—weekly diets modelled by combining the single food (sub) groups and single foods from module 3 b;

^c^Problem nutrients—nutrients that could not reach 70% of RNI

Fat, calcium and zinc were identified as problem nutrients (reaching <100% RNI) in the best-case scenario diet for the 4–5 year olds and, when testing food (sub) groups identified as nutrient dense in module 2, individually and combined (in module 3b and c), these nutrients remained as problem nutrients irrespective whether or not *Munkoyo* was included (Tables 6 and 7). However, including *Mabisi* showed improvement in calcium (≥70% RNI) with fat and zinc remaining below 70% RNI (Table 8).

**Table 6:** Shows Food based recommendations for scenario 1 with only local foods for the 4–5 year old group.

	Analysis	Nutrients as % of RNI^a^												
		Protein %	Fat %	Calcium %	Vitamin C %	Thiamin %	Riboflavin %	Niacin %	Vitamin B-6%	Folate %	Vitamin B-12%	Vitamin A RAE %	Iron %	Zinc %
	**module 2 optimized food patterns for the local**													
	Optimised Diets—Worst-case-scenario	165.8	88.1	26.7	399	81.9	76	63.5	133.3	54.9	23.4	133.2	58.5	46.6
	Optimised Diets—Best-case-scenario	291.9	73	47.8	438.2	125.6	100	141.6	188.9	115	100	163.4	100	66
	**Module 3 Phase i (Diet Without Recommendations)**													
	No Recommendation Diet—worst case scenario	115.4	21.1	4.8	46.3	50.2	31.4	36.9	74.9	22.8	3.2	0	39.2	37.3
	**Module 3 Phase ii worst case scenario nutrient level for 6 single alternative sets of recommendations**													
1	Grains & grain products 7 servings	123	22.5	4.8	46.3	50.2	31.4	36.9	74.9	22.8	3.2	0	39.5	38
2	Vegetables 21 servings	119.5	21.1	7.3	61.4	54.6	34.9	38.3	84.6	24.3	3.6	0.5	41.2	38.3
3	Rape leaves (kale) 7 servings	133	22.5	23.4	383.2	64.9	48.8	47.3	111.2	34.6	3.8	128.6	50.2	40.2
4	Cooked beans lentils peas 3 servings	148.1	21.4	8.7	46.3	55.8	34.7	37.8	79.9	47.7	3.3	0	49.4	43.1
5	Nuts seeds 5 servings	142.4	41	7.1	46.3	66	34.4	59.5	83.4	40.8	3.3	0	44.4	42.1
6	Small whole fish with bones 2 servings	163.1	29.9	8.3	47	55.1	44.5	176.2	91.2	24.5	377.2	2.1	42.2	40.7
	**Module 3 Phase iii worst case scenario nutrient level for the combined single alternative sets to form FBRs**													
	1+2+3+4+5+6^b^	259.8	53.4^c^	33.6^c^	387	92.1	69.6	210.4	143.2	79.7	378	130.7	70.7	57^c^

RNI–

^a^Recommended Nutrient Intake;

^b^Final FBRs—weekly diets modelled by combining the single food (sub) groups and single foods from module 3 b;

^c^Problem nutrients—nutrients that could not reach 70% of RNI

**Table 7:** Show Food based recommendations for scenario 2 based on local foods with *Munkoyo* added for the 4–5 year old group.

	Analysis	Nutrients as % of RNI^a^												
		Protein %	Fat %	Calcium %	Vitamin C %	Thiamin %	Riboflavin %	Niacin %	Vitamin B-6%	Folate %	Vitamin B-12%	Vitamin A RAE %	Iron %	Zinc %
	**module 2 Optimized food patterns for the local**													
	Optimised Diets—Worst-case-scenario	165.7	88.2	26.7	399	82	76.1	63.6	133.3	54.9	23.4	133.2	58.5	46.7
	Optimised Diets—Best-case-scenario	291.9	73	47.8	438.2	125.6	100	141.6	188.9	115	100	163.4	100	66
	**Module 3 Phase i (Diet Without Recommendations)**													
	No Recommendation Diet—worst case scenario	115.4	21.1	4.8	46.3	50.2	31.4	36.9	74.9	22.8	3.2	0	39.2	37.3
	**Module 3 Phase ii worst case scenario nutrient level for 6 single alternative sets of recommendations**													
1	Rape leaves (kale) 7 servings	133	22.5	23.4	383.2	64.9	48.8	47.3	111.2	34.6	3.8	128.6	50.2	40.2
2	Nuts seeds 5 servings	142.4	41	7.1	46.3	66	34.4	59.5	83.4	40.8	3.3	0	44.4	42.1
3	Vegetables 21 servings	119.5	21.1	7.3	61.4	54.6	34.9	38.3	84.6	24.3	3.6	0.5	41.2	38.3
4	Cooked beans lentils peas 3 servings	148.1	21.4	8.7	46.3	55.8	34.7	37.8	79.9	47.7	3.3	0	49.4	43.1
5	Small whole fish with bones 2 servings	163.1	29.9	8.3	47	55.1	44.5	176.2	91.2	24.5	377.2	2.1	42.2	40.7
6	*Munkoyo* 6 servings	117.3	24.6	5.8	46.4	61.1	42.1	41.6	80.3	27.8	4.6	0	39.6	41.6
7	Grains & grain products 6 servings	115.4	21.1	4.8	46.3	50.2	31.4	36.9	74.9	22.8	3.2	0	39.2	37.3
	**Module 3 Phase iii worst case scenario nutrient level for the combined single alternative sets to form FBRs**													
	1+2+3+4+5+6+7^b^	254.1	55.4^c^	35.4^c^	387.1	103.6	81.1	215.4	151.8	84.9	379.3	131	70.2	60.2^c^

RNI–

^a^Recommended Nutrient Intake;

^b^Final FBRs—weekly diets modelled by combining the single food (sub) groups and single foods from module 3 b;

^c^Problem nutrients—nutrients that could not reach 70% of RNI

**Table 8:** Shows Food based recommendations for scenario 3 based on local foods with *Mabisi* added for the 4–5 year old group.

	Analysis	Nutrients as % of RNI^a^												
		Protein %	Fat %	Calcium %	Vitamin C %	Thiamin %	Riboflavin %	Niacin %	Vitamin B-6%	Folate %	Vitamin B-12%	Vitamin A RAE %	Iron %	Zinc %
	**module 2 best food patterns for the local**													
	Optimised Diets—Worst-case-scenario	166.2	88.4	27.5	399	82	76.6	63.5	133.4	54.9	24.4	133.4	58.5	46.8
	Optimised Diets—Best-case-scenario	296.2	56.6	98.2	438.2	108.3	132	100	185.7	101.6	132.9	173.9	100	72.1
	**Module 3 Phase i (Diet Without Recommendations)**													
	No Recommendation Diet—worst case scenario	115.4	21.1	4.8	46.3	50.2	31.4	36.9	74.9	22.8	3.2	0	39.2	37.3
	**Module 3 Phase ii worst case scenario nutrient level for 7 single alternative sets of recommendations**													
1	Rape leaves (kale) 7 servings	133	22.5	23.4	383.2	64.9	48.8	47.3	111.2	34.6	3.8	128.6	50.2	40.2
2	Cooked beans lentils peas 3 servings	148.1	21.4	8.7	46.3	55.8	34.7	37.8	79.9	47.7	3.3	0	49.4	43.1
3	Small whole fish with bones 2 servings	163.1	29.9	8.3	47	55.1	44.5	176.2	91.2	24.5	377.2	2.1	42.2	40.7
4	Grains & grain products 6 servings	115.4	21.1	4.8	46.3	50.2	31.4	36.9	74.9	22.8	3.2	0	39.2	37.3
5	*Mabisi* 6 servings	154.6	37.5	49.5	46.4	55.5	72.4	38.9	84.5	27	63.8	13.6	40.3	47.3
6	Nuts seeds 5 servings	142.4	41	7.1	46.3	66	34.4	59.5	83.4	40.8	3.3	0	44.4	42.1
7	Vegetables 21 servings	119.5	21.1	7.3	61.4	54.6	34.9	38.3	84.6	24.3	3.6	0.5	41.2	38.3
	**Module 3 Phase iii worst case scenario nutrient level for the combined single alternative sets to form FBRs**													
	1+2+3+4+5+6+7^b^	291.4	68.5^c^	82.3	389.8	99.5	112.6	213.4	163.8	84.5	438.6	157.8	75.6	67^c^

RNI–

^a^Recommended Nutrient Intake;

^b^Final FBRs—weekly diets modelled by combining the single food (sub) groups and single foods from module 3 b;

^c^Problem nutrients—nutrients that could not reach 70% of RNI

The final recommendations (with most nutrients reaching ≥70% RNI in the worst case scenario of the FBRs) for the 1-3year age group included the following serves per week: Vegetables 21 servings; *Mabisi* 7 servings; Grains and grain products 7 servings; Cooked beans, lentils and peas 3 servings; Nuts, seeds and unsweetened products 4 servings; and Rape leaves 7 servings; Small whole fish with bones 2 servings (Table 5).

The final recommendations (with most nutrients reaching ≥70% RNI in the worst case scenario of the FBRs) for 4–5 year age group included the following servings per week: Vegetables 21 servings; *Mabisi* 6 servings; grains and grain products 6 servings; Cooked beans, lentils and peas 3 servings; Nuts, seeds and unsweetened products 5 servings; and Rape leaves 7 servings; Small whole fish with bones 2 servings (Table 8).

## Discussion

**Fig 2:**
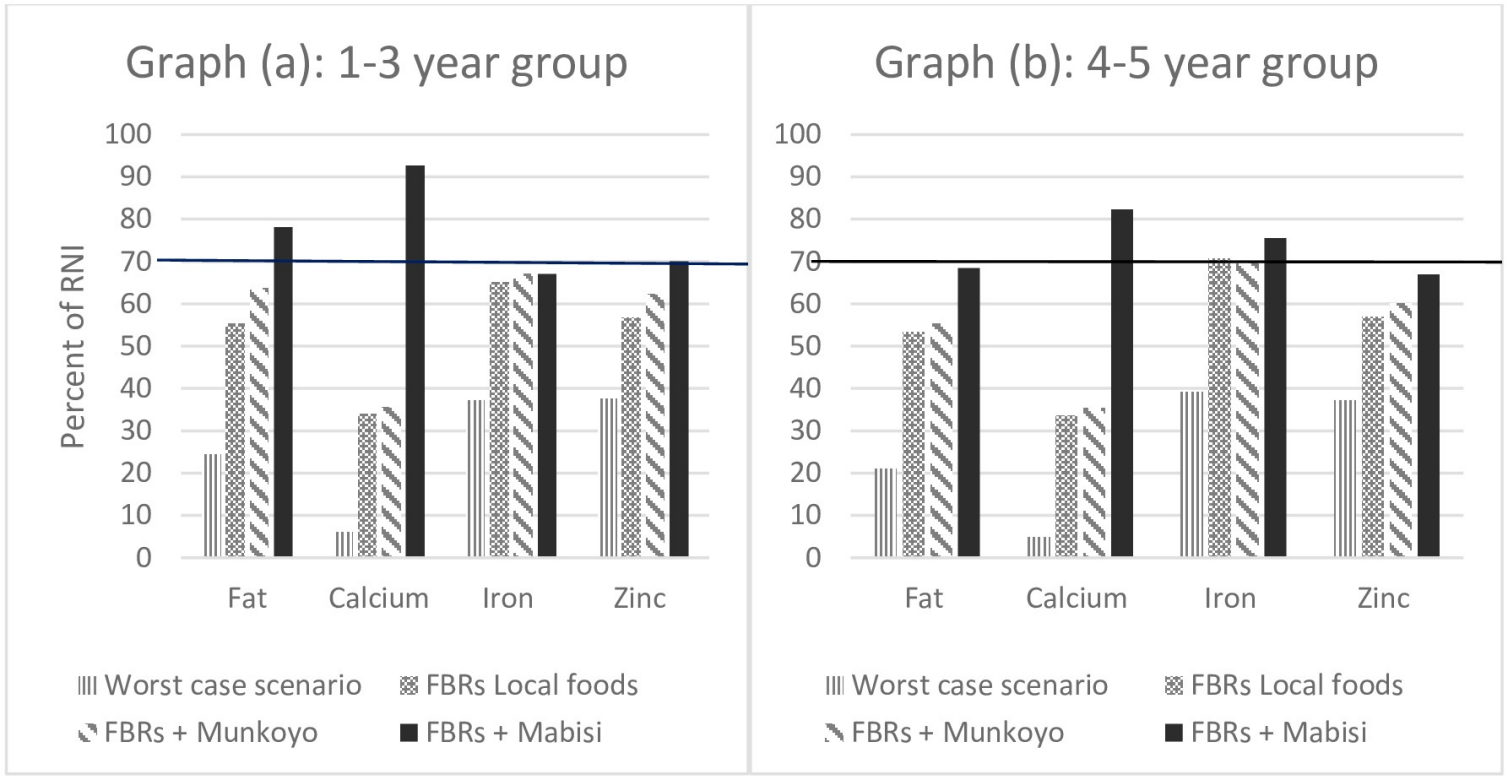
Comparison of problem nutrients for the three scenarios (FBRs with local foods only, FBRs with *Munkoyo* added and FBRs with *Mabisi* added) against the worst case scenarios for the two age groups, 1–3 years old in graph (a) and 4–5 years old in graph (b). Notes: The y-axis shows the % of RNI and the x-axis shows the problem nutrients. RNI is Recommended Nutrient Intake; FBRs is Food Based Recommendations. These are the nutrients that did not reach 70% RNI when diets were modelled for the 3 scenarios compared with the worst-case-scenario. The rest of the nutrients selected and modelled in Optifood are not depicted in this figure because they were all ≥70% RNI for all the scenarios. In the figure, the diet with *Mabisi* recorded the highest increase compared to the worst-case-scenario for the problem nutrients in both age groups).

The current analysis revealed that nutrient intake among children aged 1–5 years in Zambia can be profoundly improved through carefully selected combinations of locally available foods but will not be able to cover the nutrient requirements for fat, calcium, iron and zinc. Only the inclusion of *Mabisi (fermented milk)* and not *Munkoyo* can sufficiently improve fat, calcium, iron (for 1–3 years olds only) and zinc (for 4–5 years olds only) intake for 1–5 year olds as indicated In Fig 2.

Results from our study indicate that among 1–5 year old children in Mkushi, the intake of fat, iron, zinc and calcium is below the requirements. The identified problem nutrients in our study are consistent with other findings in Zambia. Calcium shows the lowest achievable % of RNI in the scenario’s with and without *Munkoyo* added. In Mkushi, another study also found nutrient adequacy for children between 4 and 8 years old for most nutrients except for calcium [43]. Another study in Zambia found that children between 6–18 months had inadequate nutrient intakes for iron, zinc and calcium [44]. Low consumption of animal source foods could partly explain low intake of fat, calcium, iron and zinc nutrients in our population due to a substantial increase in production and consumption of cassava, maize and vegetable oils and a corresponding decrease in that of animal source foods observed in the past decades [8]. Inclusion of *Mabisi* in the food based dietary recommendations increased the potential of adequate intake of fat, calcium, zinc and iron in our study. A review on the impact of lipid-based nutrient supplements (LNS) plus complementary foods on health, nutrition and developmental outcomes among infants and young children [45] suggested that LNS plus complementary feeding compared to no intervention is effective at improving growth outcomes and anaemia among children aged 6 to 23 months in low- and middle-income countries (LMIC) in Asia and Africa, and more effective if provided over a longer duration of time (over 12 months). A trial in Malawi comparing the developmental outcomes of 18-month-old infants who received complementary feeding for 1 year either with lipid-based nutrient supplements or micronutrient fortified corn-soy porridge [46] found that the two types of interventions have comparable developmental outcomes by 18 months of age. A number of other trials conducted on yogurt (a fermented milk product with *Lactobacillus bulgaricus* and *Streptococcus thermophilus*) consumption have linked this yogurt intake to better health outcomes including weight management, type 2 diabetes, cardiovascular, disease risk, bone health, gastrointestinal (GI) health, malnutrition, immunological parameters and overall mortality [46–54]. In many of these studies microbial diversity appeared to increase in subjects consuming yogurt and the association between a greater microbial diversity and better health conditions has been attributed to yogurt consumption. Since *Mabisi* is a fermented milk product with the potential to contribute towards meeting reference intake of fat, calcium and zinc for 1–3 year aged children and fat, calcium and iron for 4–5 year aged children, regular consumption could contribute to the improvement in nutritional status, development functions, and gut function through fermenting bacteria.

*Munkoyo* (a cereal based fermented product) did not confer significant benefits in improving nutrient intake but the children may benefit from the fermenting microbes—that include high abundance of lactic acid bacteria—in this product. Chilton et al, in a review promoting inclusion of fermented foods in dietary guidelines established that the extensive use of and nutritional and health benefits derived from the fermented foods are evident enough for recommendation of regular consumption [28].

The feasibility and generalizability of identified food based dietary recommendations need further attention and it should be evaluated before implementation; for instance whether the required behaviour change is feasible [35]. Further, the foods included in the food based dietary guidelines should be available in sufficient amounts, although availability of foods does not always result in increased consumption as other aspects influence consumption, for example accessibility and affordability [55]. Cost of the locally available foods was not taken into account when modelling the different scenario diets. While *Mabisi* is generally considered a low-cost food, it is not known whether the final FBRs (including *Mabisi*) are affordable for the parents or caretakers of the children. Generally socio-economic factors influence food choices and nutrient dense foods are in general more expensive than low nutrient dense foods [56, 57]. Furthermore, *Mabisi* should be acceptable for consumption. From our dietary intake it appeared that *Mabisi* was consumed by only three children (one from the 1–3 year age group and 2 from the 4–5 year age group). However other studies about traditional fermented foods in Zambia [30] have shown that these foods are consumed on a regular basis in all age groups and are culturally accepted. Lastly, data from only one district in Zambia (Mkushi) was used. Although generally in Zambia similar dietary patterns are observed throughout the country with main foods being cereals, roots & tubers and vegetables [58], some foods in our FBRs may not be available in all areas of Zambia. Therefore use of data from a national representative sample would probably result in FBRs that are generalizable for the whole country.

There are some limitations in this study that are acknowledged. First, we acknowledge that recall bias in dietary intake data collection using 24 hour recall is inevitable. Precautions were taken through well trained interviewers, the use of a food frequency questionnaire (FFQ) to determine the frequency of specific food items, proper calibrations and the use of pictures and plastic bowls to better visualize and estimate the serving sizes. Often in recalls, nutrition dense foods that are irregularly consumed and snacks and fruits are often forgotten. Nevertheless, we could not avoid introducing errors in the data which could have affected our results, but we tried to keep it to a minimum. We also made sure to quality control the data analysis where the lead author carried out the analysis and the second person did the same analysis and the results were comparable.

Second, FBRs were developed for children between 1 and 5 years who are not breastfed due to very few children being breastfed after 1 year of age. The World Health Organization and UNICEF recommends initiation of breastfeeding within the first hour after the birth, exclusive breastfeeding for the first six months and continued breastfeeding for two years or more, together with safe, nutritionally adequate, age appropriate, responsive complementary feeding starting at the age of sixth months [41]. Part of the target group in this study falls in this particular age range. If breastfeeding were included in the modelling for the 1–2 years old, probably the effect of *Mabisi* would have been less as the breast milk would have provided for some of the problem nutrients. However, breastfeeding after 1 year was very low, accounting for less than 5% children, and was far lower than that of the national average of 92% and 42% of all children still breastfed at age 1 year and at 2 years respectively [1]. It may be that in this region of the country breastfeeding after age 1 year is low and that changing behaviour towards extending the breastfeeding period to 2 years or beyond may be challenging due to socio-cultural issues. We think that inclusion of *Mabisi* is more acceptable than extension of breastfeeding and that also breastmilk lacks the beneficial effects of fermentation, however it is acknowledged that *Mabisi* may have a higher safety risk.

Third, we used a combination of food composition tables (FCTs) to develop the one used in this study and this may have introduced errors due to nutrient variations introduced. We did not have the nutrient composition of *Mabisi* and used the composition of sour milk that is considered similar to *Mabisi* from the FCT. We do not expect any significant differences in nutrients between *Mabisi* and sour milk as they are similar products made in the same way but with different names in different regions of the country. It is clear that fermentation reduces phytates and increases bioavailability of minerals such iron and zinc in foods and that information on iron is available in literature but it is unclear for zinc [42]. We increased zinc values modestly by 5%, similar to that of iron in the FCT taking into account the reduction of phytate that has been shown before of 15–46% after maize porridge fermentation [59] and that the increase in iron bioavailability positively correlated with zinc amounts determined from *in vitro* studies [60]. We also took into account results from *in vivo* studies in rats that have also shown a higher zinc bioavailability when fed on diets containing fermented cassava than the unfermented cassava [61]. It is remarkable that dried small whole fish species in our FCT contained very high vitamin B12 values reaching ≥300% of the RNI for vitamin B12. These values were based on data published in the African Journal of Food, Agriculture, Nutrition and Development in 2010 and a report published by Nyirenda *et al* in 2007 [9, 62]. In these studies vitamin B12 values were similar to the FCT values used in this study for dried small whole fish species. It is known that dietary patterns in low and middle income countries are mostly plant based and as animal source products are rich sources of vitamin B12, it would be expected that deficiencies in vitamin B12 exist. In a study that assessed vitamin B12 status among Zambian children under the age of five years, high levels of vitamin B12 deficiencies were found (87%) [63]. The high values for vitamin B12 in fish in our FCT might have led to an overestimation of the dietary intake of vitamin B12 for the target group. To improve the data, a direct chemical analysis of the fish consumed would have given better estimates of vitamin B12 intake.

## Conclusions

This study shows that FBRs for Zambian non-breastfed, 1–5 year old children based on only locally available foods into FBRs do not meet the required intakes for fat, calcium, iron and zinc and that the inclusion of *Mabisi* (and not *Munkoyo*) can have a major impact on the nutrient adequacy. Results in this study indicates the importance of the inclusion of *Mabisi*, a fermented milk product in the local food diet as a good additional source of nutrients for these age groups. However, to improve iron (for 1–3 years old) and zinc (for 4–5 years old) intake, alternative strategies should be found. The results from this analysis can serve as a guide for designing evidence based dietary recommendations under local conditions. Furthermore, additional data on for example cost of foods would still be needed to improve feasibility of developed FBRs.

## Supporting information

S1 Data (CSV)

S2 Data (XLSX)
